# Interesting Analyses of Articles in Foreign Journals

**Published:** 1865-01

**Authors:** 


### INTERESTING ANALYSES OF ARTICLES IN FOREIGN JOURNALS.

### *The Relief of Near-Sight without Spectacles.* By J. V. SOLOMON, Esq., F. R. C. S.

In a paper read before the British Medical Association by this gentleman, it was argued, on the method of exclusion, that the ciliary muscle must be the chief factor of those changes which produce the highest state of refraction. In myopia, or near-sight, the author said, the refraction of the dioptric media was too great; a condition which he reduced by performing an intraocular myotomy of the ciliary muscle. Mr. Solomon then noticed the serious structural changes which the opthalmoscope and dissections after death prove to take place in the optic nerve—retina, choroid, sclerotica, &c. An acquaintance with these pathological phenomena, he said, explained the great uncertainty which myopic persons experience in their powers of vision, and the subjective symptoms of congestion to which they are prone; also the not unfrequent occurrences of amblyopia, and sometimes of amaurosis.

The author contended that the infrequency of non-hereditary staphyloma posticum, unattended by atrophy of the choroid pigment, favored the opinion that the choroid, and not the sclerotica, was primarily affected in the production of elongation of the antero-posterior axis of the globe. It was evident, he remarked, from all we know of the pathology of myopia, that whatever surgical operation we may adopt for the relief of near-sight, must contain within it not only the power of decreasing the excess of refraction of the dioptric media, but of removing intraocular congestion, which is commonly connected with that state. Observations made at one of the most numerously attended provincial opthalmic hospitals, and which had extended over a period of nearly two years and a half, and embraced an experience of nearly forty operations, had led the author to the conviction that the said desiderata are more completely supplied by intraocular myotomy than by any other method. Indeed, he knew of no treatment which is easily safe and easy of execution, that exerts the same amount of curative power in cases of subacute and chronic posterior choroiditis.

After describing the ciliary muscle and his method of dividing it, Mr. Solomon said the most striking results from his plan of treatment were afforded by patients under the age of puberty. The operation enabled children to see features with as much distinctness, and at the same distance, as persons whose accommodation was normal. Prevented their acquiring the habit of dropping the lids, and of that *distrait* demeanor which myopics commonly exhibit. It also enabled them to thread a needle, pick a pin from the floor, and read a book at a moderate distance from the eye. A boy of eleven years, who could see features with distinctness at six yards only, in two years after the operation, saw them with clearness at forty yards, and the figure of a man and all his movements at five hundred. He read small pica at thirty-three inches, instead of at six, as before the operation. In adults whose eyeballs are not very wide in their transverse diameters, or their optic nerve completely encircled by a wide horned stephyloma posticum, or their choroids spoiled by extensive atrophy, a normal range for features was frequently acquired. A man, who two years ago could read small pica at nine inches and a half only, and see features distinctly at five yards, now reads at twenty-four inches, sees features at thirty yards, and trees with distinctness at a mile and a quarter.

The paper was illustrated by diagrams of the ciliary muscle, colored drawings and mathematical formulæ, illustrative of the value of the operation in the accommodation of eyes which had been treated upwards of one or two years.

*Origin of the Cow Pox.*—The Academy of Medicine of Paris has been engaged in discussing some facts of interest which have taken place at Toulouse, in France. The matter from an eruption affecting the legs of a horse was inoculated upon the teats of a cow, and produced vesicles similar to those of cow-pox; the pus, moreover, taken from this cow, having been inoculated on infants, gave rise to vaccine vesicles. It is doubtful, however, whether in this case the horse suffered from grease or some other eruptive complaint. Jenner considered that grease might be the origin of cow-pox. Experiments have therefore been made with the grease in several countries, but with variable results. It should, however, be recollected that a smith's apprentice, of Chartres, in France, who had not been vaccinated, having shod a greasy horse, presented vaccine vesicles on his hands. The pus from these being inoculated on children, gave rise to similar vesicles; but the same pus sent to the Academy of Medicine at the time, and used for inoculation, yielded no results; while, strange to say, the lymph taken from the vesicles on the apprentice's hand, being also sent to the Academy, was the origin of a series of successful inoculations.—Such was the state of the question when the facts observed at Toulouse became the subject of a report from a committee appointed for the purpose of examining into the alleged phenomena. It is this report, itself reported upon by M. Bosquet, that the Academy has been discussing.

*Galactagogues.*—M. Turnier and Dr. Highmore both report favorably on the use of galvanism as a galactagogue. In the case of the latter, the fifteenth birth, previous children not being nursed for want of milk, one of Davis and Kidder's machines was applied for about twenty minutes. This was repeated on three successive days, when there was a good supply of milk, and it was discontinued. When the instrument was first resorted to there was no milk being secreted, and the bosoms were quite flaccid. In connection with this subject it may be mentioned that the bruised leaves of the *palmi christi*, as a local application, are extensively and successfully used by European physicians for the same purpose.

